# Inclusion of stabilised rice bran in ready-to-use therapeutic food supports growth in Indonesian children with severe and moderate acute malnutrition: solutions to enhance health with alternative treatments (SEHAT), a double-blinded, randomised clinical trial

**DOI:** 10.1017/jns.2025.10074

**Published:** 2026-01-29

**Authors:** Silvia Barbazza, Annika M. Weber, Moretta D. Fauzi, Asrinisa Rachmadewi, Ririh Zuhrina, Fildzah Putri, Maiza Campos Ponce, Marinka van der Hoeven, Sonia Fortin, Rimbawan Rimbawan, Zuraidah Nasution, Puspo Edi Giriwono, Frank T. Wieringa, Damayanti D. Soekarjo, Elizabeth P. Ryan

**Affiliations:** 1 Department of Health Sciences, Faculty of Science, Vrije Universiteit Amsterdam, Amsterdam Public Health, Amsterdam, The Netherlands; 2 Department of Food Science and Human Nutrition, Colorado State University, Fort Collins, CO, USA; 3 UMR Qualisud, CIRAD, Institut Agro, IRD, Université Montpellier, Université Avignon, Université de la Réunion, Montpellier, France; 4 Child Health Department of Mohammad Hoesin Hospital, Palembang, South Sumatera, Indonesia; 5 Child Health Department of Faculty of Medicine, Sriwijaya University, Palembang, South Sumatera, Indonesia; 6 Savica Consultancy, Surabaya, Indonesia; 7 French National Research Institute for Sustainable Development (IRD), Montpellier, France; 8 Department of Community Nutrition, IPB University, Bogor, Indonesia; 9 South East Asia Food and Agriculture Science and Technology (SEAFAST) Center, IPB University, Bogor, Indonesia; 10 Department of Food Science and Technology, IPB University, Bogor, Indonesia; 11 Department of Environmental and Radiological Health Sciences, Colorado State University, Fort Collins, CO, USA

**Keywords:** Anthropometric growth, Malnutrition, Mid-upper arm circumference, Rice bran, Weight, *z*-scores

## Abstract

Ready-to-use therapeutic foods (RUTFs) are widely used to treat severe acute malnutrition (SAM) by improving key anthropometric outcomes; however, optimisation of RUTF formulations remains important to support sustained recovery. Rice bran, a novel nutrient-dense, prebiotic food ingredient, can support healthy growth. This two-arm, double-blinded, randomised controlled trial, compared the effectiveness of a locally produced RUTF with rice bran to the same RUTF without rice bran for the treatment of uncomplicated acute malnutrition in Jember, Indonesia. 200 children aged 6–59 months with SAM (WHZ < −3.0 and/or mid-upper arm circumference (MUAC) < 115 mm or having bilateral pitting oedema +/++) or approaching SAM (WHZ < −2.5) were enrolled in the study. Primary outcomes were weight, MUAC, and anthropometric *z*-scores. Linear mixed models were applied across all ages, and split by age groups (6–23 months and 24–59 months) at weeks 0, 4, 8, 12, and 16 for intention-to-treat (ITT) and per protocol analysis (PP). Children in two age groups were expected to respond differently to treatment based on their microbiome maturity. At week 4, the PP analysis revealed RUTF+rice bran treatment had significantly greater weight gain velocity (*p* = 0.02; *p* = 0.008) and MUAC velocity (*p* = 0.004, *p* = 0.03) when compared to RUTF at all ages and in the 24–59 months age group, respectively. There were no significant differences between treatment groups at time points in the other anthropometric outcomes. This investigation shows promising impact of stabilised rice bran as a prebiotic and nutrient-dense ingredient for inclusion into RUTFs that can improve child growth outcomes.

## Introduction

Globally, 45 million children under the age of five suffer from acute malnutrition.^(1)^ Severely malnourished children face a higher risk of illness and death, primarily due to acute infectious diseases.^(2,3)^ International growth standards, such as weight for height *z*-score (WHZ) < −3 and/or mid-upper arm circumference (MUAC) < 115 mm, are used to diagnose and set treatment objectives.^(4)^ The primary focus of standard treatment is early identification of wasting and outpatient therapy using ready-to-use therapeutic foods (RUTF) for patients without complications. However, without addressing the need to normalise physiological and metabolic functions, children continue to be at high risk of relapse and death.^(5)^

Indonesia has made progress towards achieving the global nutrition target for wasting, which aims to reduce the prevalence of acute malnutrition in children under five to below 5%.^(6)^ However, the national rate is currently at 8.5%,^(7)^ just lower than the region of Asia’s average of 8.9%.^(8)^ Indonesia has shown commitment to strengthening community-based treatment of severe acute malnutrition (SAM), including a manifested interest in developing and producing a local RUTF for treating SAM,^(9)^ as well as the development of national standards.^(10)^ However, locally produced RUTFs are not currently available in Indonesia, and the importation of standard treatment products is restricted. This presents major challenges for carrying out large-scale community-based treatment.

Global standards for RUTFs are primarily formulated to achieve rapid weight gain and meet short-term nutrient requirements, but do not fully address sustained malnutrition recovery, which is influenced by other factors such as seasonal household food insecurity.^(11,12)^ In parallel, there is mounting evidence that also highlights the central role of the gut microbiome in childhood growth and overall health.^(13)^ Malnourished children, even after recovery, exhibit distinct immature gut microbiota compared to their age-matched healthy counterparts,^(14)^ a factor that has been associated with increased susceptibility to malnutrition relapse.^(14–16)^ Therefore, an emerging focus exists on reformulating RUTFs using locally accessible wholefood ingredients that support recovery through multiple mechanisms. This includes the use of regionally available, seasonal, and drought-resistant crops that can strengthen household food security^(17)^ while also benefiting overall health and the gut microbiome.^(15)^ RUTF formulations have incorporated various new ingredients such as legumes/pulses, oats, green banana powder, and chickpea powder.^(18–20)^ These crops contribute nutrient dense food options that enhance household food security and provide prebiotic fibres alongside essential amino acids, micronutrients, and minerals. Importantly, their inclusion supports agricultural sustainability, while promoting gut microbiome maturation and improved anthropometric outcomes during treatment^(18,19)^ and reducing relapse risk post-treatment.^(21)^

Rice bran, the outer layer of brown rice, is an underutilised human food ingredient, and is often discarded or used for animal feed.^(22)^ Rice bran contains a suite of prebiotic fibres, essential fatty acids, amino acids, micronutrients, and phytochemicals.^(23–25)^ Due to the unique nutrient density of rice bran, alongside the local accessibility and affordability that can address household food security, there is great potential for incorporating this novel ingredient in local RUTF development, especially in countries where rice is grown as a staple crop. Rice bran can add value to the food system by transforming a widely available milling by-product into a nutrient-dense resource that can improve human and animal nutrition, reduce food waste, enhance the sustainability and profitability of rice production, and create new economic opportunities through its use in foods and feeds. Previously, rice bran has demonstrated the capacity to prevent enteric pathogens such as *Salmonella enterica Typhimurium*,^(26)^ norovirus,^(27)^ and rotavirus,^(28)^ as well as has exhibited the ability to enhance mucosal barrier function and promote the growth of native gut probiotics.^(29)^ Recently, rice bran was found to be acceptable in local diets in rural Guatemala and led to improvements in meeting daily nutrient requirements for children and mothers at risk for malnutrition.^(30)^ Furthermore, in clinical trials in Nicaragua and Mali, rice bran consumption increased linear growth^(31)^ and improved gut-microbiota diversity in infants at risk for malnutrition.^(32)^ Rice bran, however, has not yet been applied in the context of acute malnutrition treatment.

Consistent with the published clinical protocol,^(33)^ we aimed to compare the effectiveness of a locally produced RUTF^(34)^ that has been previously tested for its acceptability and efficacy^(35)^ to an RUTF with 5% rice bran for eight weeks of treatment in children with acute malnutrition using a double-blinded, randomised clinical trial study design. Here, we report the primary outcomes, including changes in weight, mid-upper arm circumference (MUAC), weight-for-height *z*-score (WHZ), height-for-age *z*-score (HAZ), weight-for-age *z*-score (WAZ), at baseline (week 0), during treatment (weeks 4 and 8), and post-treatment (weeks 12 and 16).

## Methods

### Study design

This study was a double-blinded, randomised, controlled clinical trial comparing a locally produced RUTF to the same RUTF with rice bran (RUTF+rice bran) for the treatment of acute malnutrition. The objective of this study was to determine the efficacy of RUTF+rice bran compared to RUTF alone in the community-based treatment of acute malnutrition in children aged 6–59 months. The primary outcomes in this study include changes in weight, MUAC, WHZ, HAZ, and WAZ. A detailed description of all study procedures is provided in the previously published protocol.^(33)^

### Participants

The trial was conducted in Jember District of East Java Province, Indonesia, from December 2022 to August 2023. Initially, children were identified through screening for malnutrition using the health centre’s electronic database. Eligible children were invited for screening at the nearest health post or their homes, following parental consent. Children between 6–59 months of age with uncomplicated SAM (WHZ < −3.0 and/or MUAC < 115 mm or having bilateral pitting oedema +/++) or approaching SAM (WHZ < −2.5) were eligible for enrolment. Children approaching SAM were included for feasibility of recruitment. Children meeting these anthropometric criteria were assessed for specific exclusion criteria, as previously described,^(33)^ including failing the appetite test, having a known allergy to one of RUTF ingredients, having a body weight < 4.0 kg, being diagnosed with medical complications, severe anaemia, tuberculosis (TB), HIV positive status or severe oedema (+++), having any congenital disorder that interferes with normal nutrient intake, having chronic conditions, being a sibling of a study participant or being enrolled in another clinical trial.

### Randomisation and blinding

Two hundred children were randomly allocated to either intervention group using a computer-generated list with a block size of 8, two codes per treatment group, making up four codes. Randomisation was performed prior to the appetite test, based on children’s age group (6–23 months and 24–59 months) by a principal investigator who did not interface with participants or the field teams to avoid interfering with study blinding. The intervention was blinded to researchers, caregivers, and field teams until the study’s completion, including primary outcome analysis. The products were packaged identically and labelled with a code and flavour.

### Intervention

Children were randomly assigned to one of the two arms for the study duration:

Arm 1: RUTF: RUTF with no rice bran

Arm 2: RUTF+rice bran: RUTF with 5% rice bran

The use of a standard imported peanut-based RUTF was not included as a treatment arm due to demonstrated low acceptability in Indonesia and the region,^(35–38)^ and inclusion was deemed not ethical by the Ethical Committee. The RUTF and the RUTF+rice bran were produced locally by the Southeast Asia Food and Agriculture Science and Technology (SEAFAST) Centre and the Department of Community Nutrition, Bogor Agricultural University in Bogor, Indonesia. The original recipes of both RUTFs are based on the results of a previous study on development of the product^(34)^ and on acceptability and efficacy study of the product that was conducted in Bogor, Indonesia.^(35)^ The quantity of vitamin and mineral premix included was formulated to meet the nutritional requirements for RUTF as specified by the CODEX guidelines.^(39)^ Details on the RUTF ingredients and nutritional profile have been previously published^(33–35)^ (and available in Supplemental Table 1). Briefly, the RUTF consisted of a wafer filled with a paste containing palm oil, whole and skim milk powder, peanut butter, sugar, whey protein concentrate, wheat and rice flour, a premix of vitamins and minerals, maltodextrin, and either vanilla or cocoa powder. In RUTF+rice bran, maltodextrin, skim milk powder, oil, sugar, and wheat flour were partially replaced with 5% rice bran (rice bran nutritional data are described in Supplemental Table 2). The stabilised rice bran was supplied by Colorado State University (purchased from Stabil Nutrition, St. Louis, MO). For each treatment arm, RUTFs were available in a chocolate or vanilla flavour, with the option to switch flavours after the first four weeks of treatment to increase participant compliance. Each sachet (seven wafer rolls) guaranteed 500 Kcal. The safety of the locally produced RUTFs was assured by following the international standards developed by the World Health Organization (WHO) and the Food and Agriculture Organization (FAO) Codex Alimentarius on Guidelines for Ready-to-use Therapeutic Foods CXG 95-2022 and the Recommended International Code of Hygienic Practice for Foods for Infants and Children CAC/RCP 21-197. The production facility, PT Javaindo Maju Sejahtera, is certified by Indonesian Government bodies regulating Good Manufacturing Practices (GMP) and Halal standards. The final products underwent rigorous microbiological and chemical food safety testing.^(34)^

### Monitoring and follow-up

As previously described,^(33)^ enrolment into the study was confirmed when children passed screening eligibility criteria, medical examination, appetite test, and caregiver’s informed consent was obtained. The study consisted of eight weeks of treatment and eight weeks of follow-up. Treatment followed the guidelines from the Ministry of Health of Indonesia,^(40)^ which included a 5-day course of Amoxicillin at enrolment, provided by the health centres and administered by the field team. Vitamin A was given by the health centre, and the field team completed deworming if the child had not yet received this treatment. At the baseline visit, fieldworkers supplied a 1-week supply of RUTF based on the child’s body weight, according to WHO standards^(41)^ (Supplemental Table 3), and provided instructions on how to give the RUTF to the child. Caregivers were asked to record the child’s daily RUTF consumption and keep any leftover RUTF. Fieldworkers visited participant homes weekly during the eight weeks of RUTF treatment. In the weekly home visits, fieldworkers provided a weekly supply of RUTF, collected measures of weight, MUAC, and oedema, recorded any side effects of the RUTF, and tracked morbidity using a weekly recall questionnaire. Fieldworkers collected records of the child’s RUTF intake to calculate daily RUTF consumption, and any unconsumed RUTF was brought back to basecamps, where it was weighed and recorded. Measures of height and other anthropometric measures were also collected at week 4 and week 8. Treatment ended for all participants after eight weeks. Children who did not gain weight after the first 4 weeks of treatment, did not consume RUTF for >4 days in a week for two consecutive weeks, or developed a medical complication/major reaction were withdrawn from the study and referred to the local health clinic. Post-treatment follow-up visits were conducted at weeks 12 and 16, where weight, height, MUAC, and oedema were collected. Full details of these procedures have been previously published.^(33)^ The study ended for all participants at week 16.

### Statistical analysis

#### Sample size

As previously stated,^(33)^ the sample size was calculated based on the WHO weight gain requirement of 5g/kg BW/day,^(41,42)^ allowing a 20% difference between treatment arms, SD of 1.5 g/kg BW, 80% power at a 5% significance level and accounted for a 15% dropout rate. If children fail to reach WHO weight gain requirements and gain only 2g/kg BW/day, as in previous studies in Indonesia,^(40,43)^ keeping the other parameters constant, 75 children per group would allow a difference of 30% between treatment groups. Based on these parameters and previous similar studies, 200 children total were recruited.

#### Data management

All data was recorded by fieldworkers either on paper or in the KoboToolbox application on an offline tablet. Data was monitored for accuracy and completeness and was verified by the field supervisors. After the field supervisor’s approval, the data was uploaded to KoboToolbox’s online cloud. Upon completion of the study, data was exported from KoboToolbox and cleaned in R (version 4.2.2) for analysis.

#### Statistical analysis

Data was analysed by blinded investigators. All data was analysed using R statistical^(44)^ software (v4.3.2). Missing data was not imputed. Child age, sex, and anthropometric data were used to calculate WHZ, HAZ, and WAZ using the WHO Child Growth Standards from the ‘zscorer’ package in R^(45)^ (v0.3.1). Baseline numerical data were tested for normality using the Shapiro-Wilks test in R. All numerical data was not normally distributed (*p* < 0.05). The Wilcoxon rank sum test was applied to test for differences in baseline data by treatment group. Pearson’s Chi-Square test was used for all categorical data. Continuous baseline data are presented as medians (IQR), and categorical variables are presented as *n* (%).

Statistical outcomes were analysed in R statistical software^(44)^ (v4.3.2) and employed the lme4 (v1.1.35.5), emmeans (v1.10.2), broom (v1.0.6), and tidyverse (v2.0.0) packages.^(46–49)^ Primary analysis outcomes used an intention-to-treat (ITT) analysis, which included all children enrolled in the study. A linear mixed model to account for repeated measures was fit to assess the incremental growth (weight, WHZ, WAZ, HAZ, and MUAC) between the two RUTFs (RUTF, RUTF+rice bran). The models were analysed with week × treatment interaction, sex and age of the children were included as fixed effects, and child ID as a random effect. The model was applied to all participants (6–59 months of age) and for each age group (children aged 6–23 months and 24–59 months). Data from the 6–23 months and 24–59 months were analysed separately, as children were expected to differ in their anthropometric kinetics as a response to treatment.^(15)^ Model assumptions were tested by assessment of residuals. Results are presented as means and standard error (SE). Statistical significance of *p* < 0.05 for the main effects and tests of interaction was applied, and further comparisons were run also when *p* > 0.05. Pairwise comparisons were employed to compare mean growth outcomes by treatment for each week and Bonferroni adjustments for multiple comparisons were applied.

In this clinical trial, due to Indonesian policy and ethical considerations, children who did not gain weight by week 4 were excluded from the trial due to failing therapy and were counted as dropouts. Therefore, while typically, the ITT is preferred to minimise bias, as these dropouts were directly related to an outcome of interest (weight gain), a per-protocol (PP) analysis was additionally performed to avoid sampling bias. The PP included children who completed all major time points (weeks 0, 4, 8, 12, and 16), and did not include children who dropped out (or were transferred out of the program and referred to local health centres if they did not gain weight after 4 weeks of RUTF treatment). As there were no significant differences in the number of children who dropped out between the two treatment arms, we considered no bias to be introduced by the PP analysis. Importantly, the outcomes analysed through the PP were velocities, or difference from baselines in weight, MUAC and *z*-scores (WHZ, WAZ, HAZ). Importantly, children were excluded when at week 3 they had the same weight, or less, as at week 0, therefore, weight and height for week 4 was not captured. As at least two time points are needed for calculations of velocity, this further justified the choice for this type of PP analysis. A linear mixed model to account for repeated measures was fit to assess these velocities (weight, MUAC, WHZ, HAZ, and WAZ) between the two RUTFs (RUTF, RUTF+rice bran). The models were analysed with week × treatment interaction, sex and age of the children were included as fixed effects, and child ID as a random effect. The model was applied to All Ages (6–59 months of age), as well as for each age group (children aged 6–23 months, and children aged 24–59 months). Results are presented as mean and standard error (SE). Statistical significance of *p* < 0.05 for main effects and tests of interaction was applied, and further comparisons were run also when *p* > 0.05. Pairwise comparisons were employed to compare mean growth outcomes by treatment for each week, and Bonferroni adjustments for multiple comparisons were applied.

#### Ethical considerations

The trial is registered on clinicaltrials.gov NCT05319717 and has Colorado State University IRB approval (IRB #1823, OHRP FWA00000647), as well as approval in Indonesia by the Medical and Health Research Ethical Committee (MHREC) at the Faculty of Medicine, Public Health and Nursing, Universitas Gadjah Mada in Yogyakarta, Indonesia Ref. No.: KE/FK/0546/EC/2022 and KE/FK/0703/EC/2023. The study adhered to the ethical standards of the Declaration of Helsinki Written informed consent was obtained from caregivers prior to any study procedures. Field workers were Indonesians, with a minimum education of an associate degree (D3) in Nutrition, had experience in research and fieldwork, and lived in Jember for the duration of the study. Informed consent forms were requested to be signed by both parents (father and mother) to minimise dropouts by asking for the father’s involvement. The procedures and informed consent forms were approved by the ethics committee.

## Results

**Figure 1.**
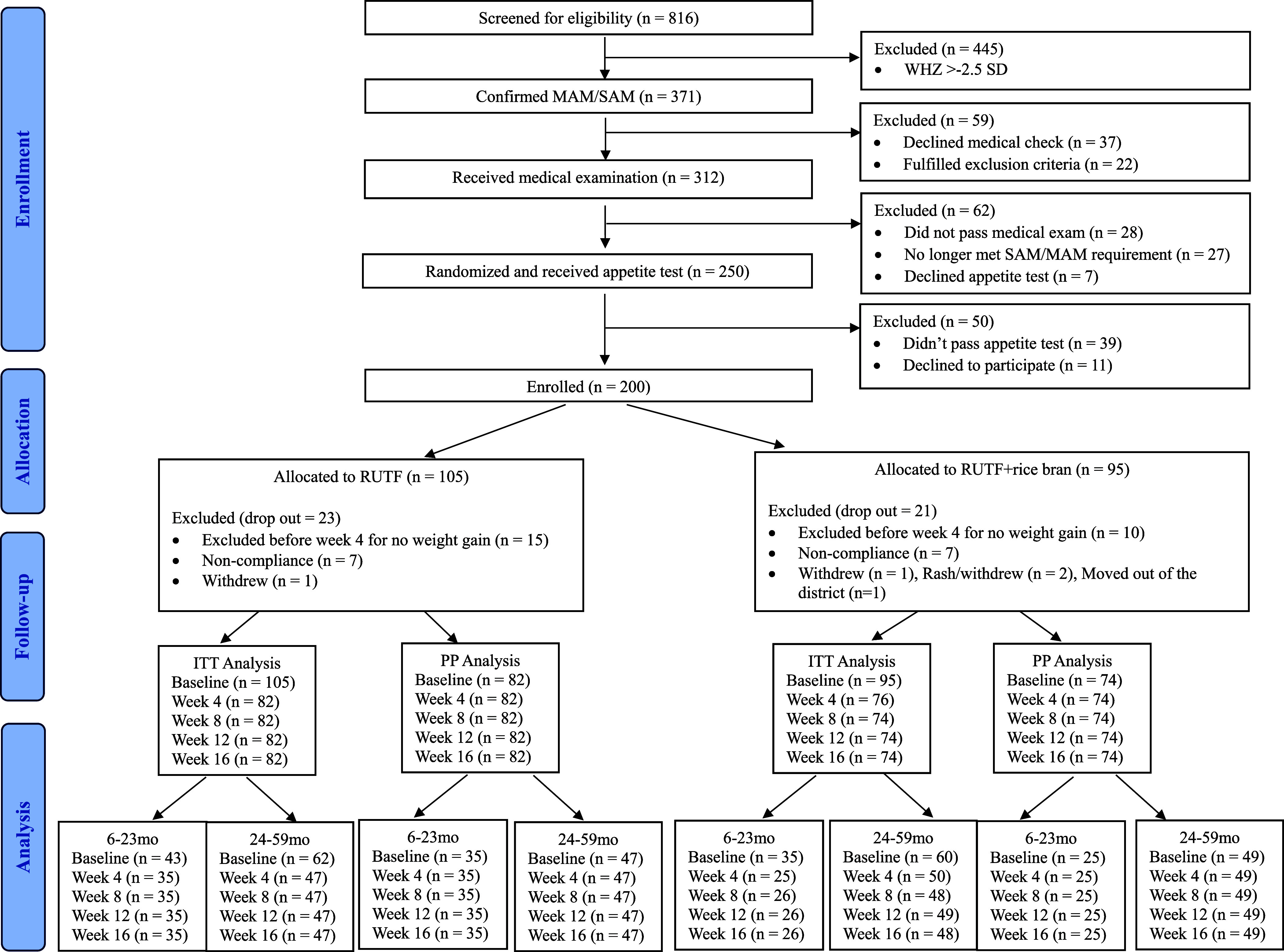
CONSORT flow. MAM: Moderate acute malnutrition (WHZ < −2.5). SAM: Severe acute malnutrition (WHZ < −3.0 and/or MUAC < 115 mm or having bilateral pitting oedema +/++).

From December 1^st^, 2022, to April 11^th^, 2023, 816 children were screened for study eligibility. After the eligibility assessment, 200 children were enrolled and randomly assigned to a treatment arm (RUTF, *n* = 105; RUTF+rice bran, *n* = 95). The number of participants differs between the two groups because randomisation was done before the appetite test, resulting in the exclusion of some children who did not meet the test’s criteria. A total of 25 children dropped out at week 4 due to failure to gain weight and were referred to local health clinics as per protocol, and 18 children dropped out due to non-compliance or other reasons. There was no significant difference in total dropouts between the two groups. In total, 156 children completed the 16-week study (RUTF, *n* = 82; RUTF+rice bran, *n* = 74; Figure 1).

Participant characteristics are shown in Table 1. At baseline, participant sociodemographic, anthropometric, and medical characteristics were similar. Compliance to study product was 20.94% and 21.23% on average for RUTF and RUTF+rice bran, respectively over the 8-week study period (Supplemental Table 4). Both RUTFs were well tolerated, and no adverse events were reported. Although recovery was not a primary outcome, graduation from acute malnutrition, defined as SAM or MAM at enrolment graduated to MAM or fully recovered (defined as: WHLZ > = −2 and or MUAC > = 12.5) at week 8, 12 or 16, was 41% and 49% in the RUTF and RUTF+rice bran treatment arms, respectively (Supplemental Table 5. There were no significant differences between treatments.

**Table 1.** Participant baseline characteristics

Characteristics	6–23 months of age		24–59 months of age	
	RUTF, *n* = 43	RUTF+rice bran, *n* = 35	RUTF, *n* = 62	RUTF+rice bran, *n* = 60
Sociodemographic characteristics				
Female, *n* (%)	17 (40)	13 (37)	28 (45)	25 (42)
Age, months	16.6 (12.4, 20.0)	20.3 (15.6, 21.9)	40 (29, 51)	42 (36, 51)
Childbirth weight (g)	2,670 (2,200, 3,095)	2,700 (2,440, 2,973)	2,700 (2,254, 3,000)	2,700 (2,475, 2,900)
Low birth weight, *n* (%)	14 (33)	10 (29)	21(34)	15(25)
Vaginal birth, *n* (%)	34 (79)	29 (83)	54 (87)	47 (78)
Currently breastfeeding, *n* (%)	31(72)	20 (57)	7(11)	5(5.3)
Respondent main caregiver of child, *n* (%)	43 (100)	33 (94)	62(100)	58(97)
Age of biological mother	28 (23, 31)	30 (28, 36)	29.5 (25.3, 34.8)	30.0 (26.0, 33.3)
Mother education, years	12 (9, 12)	10 (6, 12)	9 (6, 12)	9(6,12)
Age of biological father	31 (27, 37)	35 (31, 40)	35 (30, 40)	34 (30, 39)
Father education, years	12 (9, 12)	9 (6, 12)	9 (6, 12)	9(6,12)
Hand washing facility in the house, *n* (%)	39 (91)	29 (83)	55 (89)	52 (87)
Animals indoors, *n* (%)	7 (16)	10 (29)	15 (24)	14 (23)
Ethnicity, *n* (%)				
Jawa	19 (44)	13 (37)	27 (44)	20 (33)
Madura	12 (28)	7 (20)	17 (27)	21 (35)
Campuran (Jawa-Madura)	11 (26)	14 (40)	18 (29)	18 (30)
Religion: Islam, *n* (%)	43 (100)	35 (100)	62 (100)	60 (100)
Anthropometric characteristics				
Mid-upper arm circumference, cm	12.50 (11.90, 12.75)	12.40 (11.95, 12.85)	13.00 (12.63, 13.50)	13.00 (12.58, 13.53)
Weight, kg	6.90 (6.35, 7.40)	7.20 (6.50, 7.75)	9.45 (8.23, 10.90)	9.55 (8.80, 10.80)
Length or height, cm	72.5 (69.2, 75.4)	74.2 (70.3, 77.3)	87 (82, 94)	89 (84, 92)
SAM, < −3 weight-for-height *z* score, *n* (%)	27 (63)	17 (49)	20 (32)	25 (48)
Weight-for-height *z* score	−3.10 (−3.30, −2.80)	−2.98 (−3.32, −2.75)	−2.91 (−3.21, −2.77)	−2.97 (−3.21, −2.77)
Height-for-age *z* score	−2.77 (−3.17, −1.74)	−2.64 (−3.33, −1.92)	−2.66 (−3.20, −2.10)	−2.78 (−3.32, −2.28)
Weight-for-age *z* score	−3.51 (−3.78, −3.06)	−3.43 (−4.05, −3.15)	−3.45 (−3.86, −3.13)	−3.64 (−3.82, −3.22)
Medical characteristics at enrolment				
Oedema, *n* (%)	1 (2.3)	0 (0)	0 (0)	0 (0)
Complete immunisation for age, *n* (%)	42 (98)	35 (100)	61 (98)	56 (93)
Fever, *n* (%)	5 (12)	2 (5.7)	5 (8.1)	8 (13)
Cough, *n* (%)	9 (21)	7 (20)	15 (24)	13 (22)
Diarrhoea, *n* (%)	1 (2.3)	1 (2.9)	2 (3.2)	0 (0)
Vomiting, *n* (%)	3 (7)	4 (11)	2 (3.2)	1 (1.7)
Rash, *n* (%)	5 (12)	0 (0)	1 (1.6)	1 (1.7)
Haemoglobin, g/dL	10.80 (10.00, 11.23)	10.80 (10.30, 11.75)	11.40 (10.80, 11.90)	11.50 (10.35, 11.95)

### Intention-to-treat analysis

**Figure 2.**
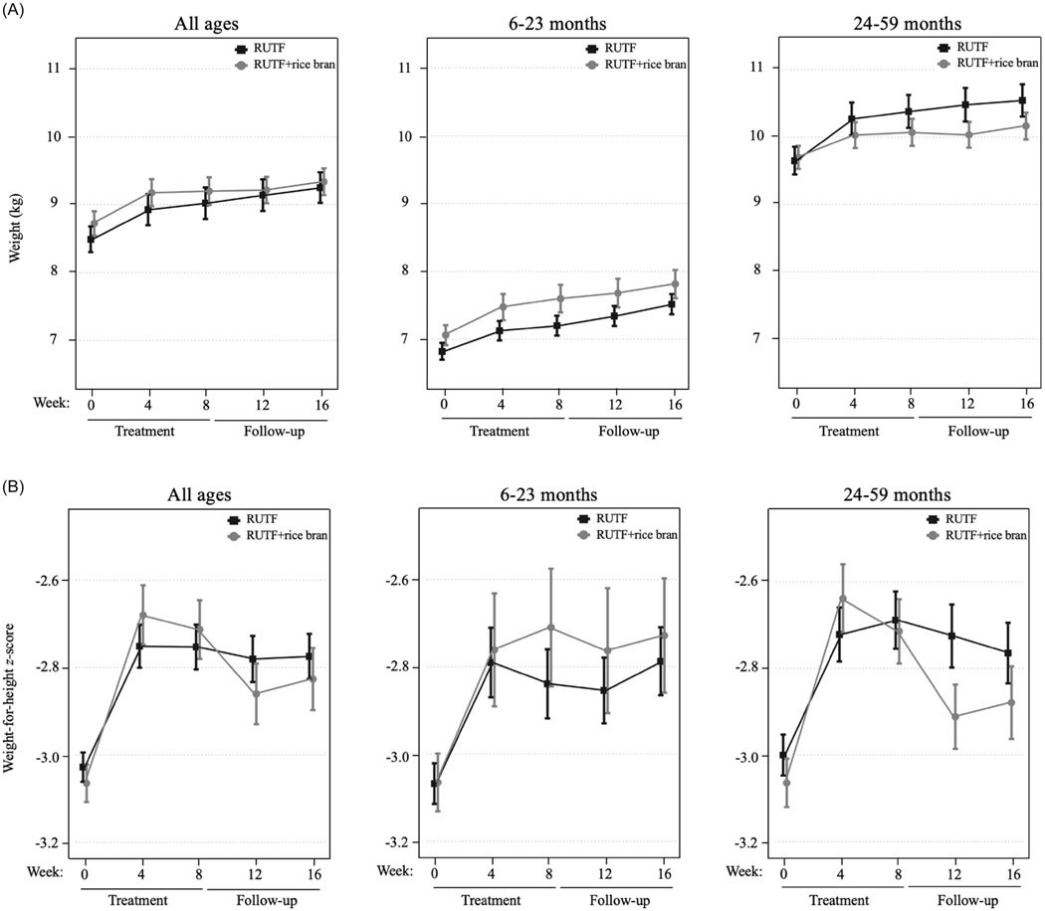
Intention-to-treat analysis of mean and standard error shown for the RUTF and RUTF+rice bran treatment arms at baseline (week 0), during treatment (weeks 4 and 8) and follow-up (weeks 12 and 16). Graphs represent data for all ages, and split age groups (6–23 months and 24–59 months) for weight (kg) (**A**) and weight-for-height *z*-scores (**B**). For between-arm comparisons (mixed linear model, pairwise comparisons with Bonferroni adjustment) at each time point.

An intention-to-treat (ITT) design was applied for primary outcome analyses, which included all children enrolled in the trial at baseline. Figure 2 illustrates the primary study outcomes of weight and WHZ, which had evidence of an interaction between RUTF treatment and week from the linear mixed model. There was an interaction between RUTF treatment and week on weight gain for all ages (*p* = 0.020, Supplemental Table 6) as well as in the 24–59 month age group (*p* = 0.032, Supplemental Table 6), though there were no significant differences between treatments at any specific time point (Supplemental Table 7). There was a significant interaction between RUTF treatment and week on WHZ, only in the 24–59 month group (*p* = 0.014, Supplemental Table 6), but no significant differences were found between treatments at any specific time point (Supplemental Table 7). There was no evidence of interaction between treatment and week on the outcomes of WAZ, HAZ or MUAC (*p* > 0.05, Supplemental Table 6), nor any significant differences between treatments at any time point (Table 2). The largest increase in WAZ occurred in the first four weeks of treatment in both arms for all age groups, and RUTF demonstrated a greater effect on incremental improvements, though not significant. The study found that the impact of treatment on HAZ varied by age group. In the 6–23 month age group, HAZ increased in both treatment groups during the first four weeks of treatment, with a stronger effect in the RUTF+rice bran group. MUAC increased during treatment in both arms, with an overall greater MUAC at week 4 and week 8 in the RUTF+rice bran arm.

**Table 2.** Intention to treat analysis for outcomes of weight-for-age *z*-score, height-for-age *z*-score and mid-upper arm circumference, and per protocol analysis of weight gain velocity and mid-upper arm circumference velocity between RUTF and RUTF+rice bran and split by age groups

Intention to treat analysis	Week	All ages			6–23 months of age			24–59 months of age		
		RUTF	RUTF+ rice bran	*p*-value^a^	RUTF	RUTF+ rice bran	*p*-value^a^	RUTF	RUTF+ rice bran	*p*-value^a^
Weight-for-age *z*-score	Baseline	−3.48 (0.05)	−3.59 (0.06)	0.60	−3.49 (0.08)	−3.57 (0.10)	1	−3.48 (0.07)	−3.61 (0.08)	0.50
	4	−3.23 (0.07)	−3.36 (0.08)	1	−3.25 (0.11)	−3.32 (0.15)	1	−3.22 (0.08)	−3.38 (0.09)	1
	8	−3.26 (0.07)	−3.38 (0.08)	1	−3.32 (0.11)	−3.34 (0.16)	1	−3.22 (0.08)	−3.40 (0.09)	1
	12	−3.26 (0.07)	−3.46 (0.08)	0.55	−3.30 (0.10)	−3.39 (0.15)	1	−3.23 (0.09)	−3.50 (0.08)	0.25
	16	−3.25 (0.06)	−3.44 (0.08)	0.65	−3.25 (0.11)	−3.37 (0.15)	1	−3.24 (0.08)	−3.47 (0.09)	0.60
Height-for-age *z*-score	Baseline	−2.60 (0.09)	−2.78 (0.10)	0.60	−2.58 (0.15)	−2.76 (0.18)	1	−2.60 (0.12)	−2.80 (0.12)	0.75
	4	−2.46 (0.10)	−2.75 (0.11)	0.70	−2.52 (0.16)	−2.67 (0.22)	1	−2.42 (0.14)	−2.80 (0.13)	0.85
	8	−2.51 (0.10)	−2.76 (0.11)	1	−2.60 (0.16)	−2.73 (0.20)	1	−2.45 (0.14)	−2.78 (0.14)	1
	12	−2.47 (0.10)	−2.74 (0.11)	1	−2.55 (0.15)	−2.76 (0.21)	1	−2.40 (0.14)	−2.74 (0.14)	1
	16	−2.46 (0.10)	−2.75 (0.11)	0.85	−2.54 (0.16)	−2.76 (0.20)	1	−2.40 (0.14)	−2.74 (0.14)	1
Mid-upper arm circumference, cm	Baseline	12.77 (0.07)	12.78 (0.08)	1	12.33 (0.11)	12.30 (0.12)	1	13.07 (0.07)	13.05 (0.08)	1
	4	13.19 (0.09)	13.35 (0.10)	1	12.72 (0.12)	12.75 (0.16)	1	13.53 (0.10)	13.65 (0.10)	1
	8	13.26 (0.09)	13.39 (0.10)	1	12.78 (0.12)	12.84 (0.17)	1	13.61 (0.10)	13.69 (0.11)	1
	12	13.24 (0.08)	13.29 (0.09)	1	12.77 (0.12)	12.82 (0.17)	1	13.59 (0.09)	13.53 (0.10)	1
	16	13.24 (0.08)	13.28 (0.10)	1	12.80 (0.13)	12.83 (0.18)	1	13.58 (0.09)	13.52 (0.11)	1

Per protocol analysis	Week	RUTF	RUTF+ rice bran	*p*-value^a^	RUTF	RUTF+ rice bran	*p*-value^a^	RUTF	RUTF+ rice bran	*p*-value^a^
Weight gain velocity, g/kg/day	0−4	1.52 (0.10)	1.82 (0.16)	**0.02**	1.80 (0.18)	1.94 (0.27)	1	1.31 (0.11)	1.76 (0.19)	**0.008**
	0−8	0.96 (0.06)	1.11 (0.09)	0.44	1.10 (0.11)	1.31 (0.14)	0.85	0.86 (0.07)	1.00 (0.11)	1
	0−12	0.82 (0.05)	0.76 (0.06)	1	0.99 (0.07)	1.01 (0.11)	1	0.69 (0.05)	0.62 (0.07)	1
	0−16	0.75 (0.04)	0.70 (0.05)	1	0.97 (0.07)	0.93 (0.08)	1	0.59 (0.04)	0.57 (0.05)	1
Mid-upper arm circumference velocity, mm/week	0−4	1.04 (0.11)	1.42 (0.15)	**0.004**	1.04 (0.11)	1.42 (0.15)	1	1.12 (0.14)	1.52 (0.19)	**0.03**
	0−8	0.61 (0.06)	0.77 (0.08)	0.67	0.61 (0.06)	0.77 (0.08)	1	0.66 (0.07)	0.79 (0.10)	1
	0−12	0.39 (0.04)	0.43 (0.05)	1	0.39 (0.04)	0.42 (0.05)	1	0.42 (0.05)	0.40 (0.06)	1
	0−16	0.30 (0.03)	0.32 (0.04)	1	−0.01 (0.03)	−0.06 (0.03)	1	−0.02 (0.04)	−0.10 (0.04)	1

Data are reported as means (SE). Statistical significance level *p <* 0.05.

The number of children analysed per week, per treatment arm in the ITT analysis was: at baseline, RUTF *n*=105, RUTF+rice bran *n* = 95; week 4, RUTF *n* = 82, RUTF+rice bran *n* = 76; week 8, RUTF *n* = 82, RUTF+rice bran *n* = 74; week 12, RUTF *n* = 82, RUTF+rice bran *n* = 74; week 16, RUTF *n* = 82, RUTF+rice bran *n* = 74.

The number of children analysed per week, per treatment arm in the PP analysis was: at baseline, and weeks 4, 8, 12 and 16, RUTF *n* = 82, RUTF+rice bran *n* = 74.

^a^Pairwise comparisons with Bonferroni adjustment. Groups were compared using a mixed linear model. The linear mixed model fixed effects include treatment, week (0, 4, 8, 12, 16), treatment*week interactions, age, and sex.

### Per-protocol analysis

The study protocol required enrolled children who did not gain weight by week 4 to withdraw from the study due to ethical considerations and were referred to a local health centre for follow-up. This requirement directly impacted reporting on primary outcomes, and therefore, a PP analysis was also performed to verify outcomes were not biased and to explore growth velocities between treatment groups.

Table 2 demonstrates the weight gain velocity (g/kg/day) and MUAC velocity (mm/week) calculated as the average over 4, 8, 12, and 16 weeks. The greater weight gain velocity was observed in the first four weeks of the study intervention, where RUTF+rice bran had significant increases in weight gain as compared to RUTF in all ages group (*p* = 0.02). When splitting by age groups, this difference was still significant in the 24–59 months group in the first four weeks of treatment (*p* = 0.008). Weight gain velocity decreased in both treatment groups and for all age groups after the first four weeks of treatment. Similarly, MUAC velocity was the greatest in the first four weeks of the intervention. In these first four weeks of the trial, children treated with the RUTF+rice bran had significantly greater MUAC velocity (*p* = 0.004) than those in the RUTF group in all ages group. When split by age groups, this difference remained significant in the 24–59 months group during the initial four months of treatment (*p* = 0.03).

**Figure 3.**
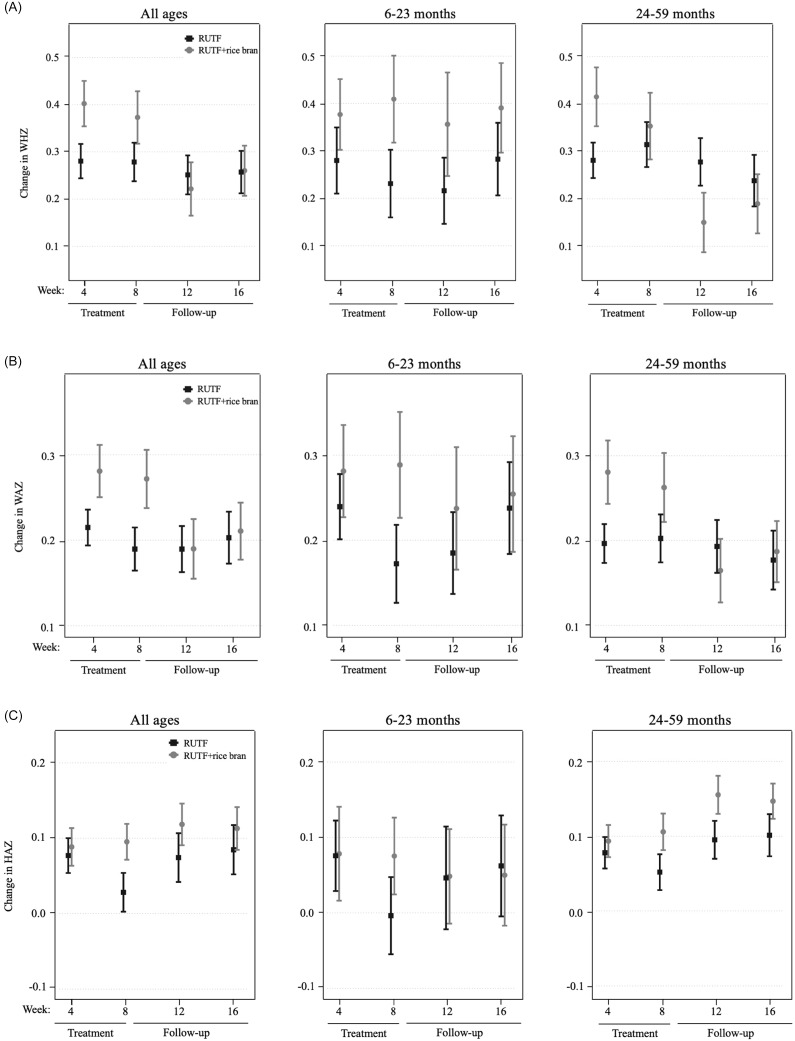
Per protocol velocity analysis of mean and standard error of the RUTF and RUTF+rice bran treatment arms expressed as change from baseline (week 0). Graphs represent data for all ages, and split age groups (6–23 months and 24–59 months) for change in weight-for-height *z*-score (**A**), weight-for-age *z*-score (**B**), and height-for-age *z*-score (**C**). For between-arm comparisons (mixed linear model, pairwise comparisons with Bonferroni adjustment) at each time point.

Changes in WHZ (Figure 3, panel A) in the all ages group were greater in the RUTF+rice bran group during treatment, though not significant. In the 6–23 month group, RUTF+rice bran had greater changes in WHZ than RUTF both during and after treatment. In the 24–59 month group, the RUTF+rice bran arm exhibited a greater change in WHZ during the initial four weeks of treatment, though not significant (Supplemental Table 8). As expected, changes in WAZ were greatest during the first 4 weeks of treatment in all groups (Figure 3, panel B). Overall, the RUTF+rice bran arm tended to have greater WAZ changes, though not reaching statistical significance (Supplemental Table 8). When examining HAZ changes (Figure 3, panel C), in the all ages group and children aged 6–23 months, there was very little change in height compared to baseline. On the other hand, there was a greater change in HAZ in the children aged 24–59 months, which was larger in the RUTF+rice bran arm (Supplemental Table 8).

## Discussion

This clinical trial investigated the effect of a locally produced RUTF+rice bran compared to the same RUTF without rice bran on weight, MUAC, WHZ, WAZ, and HAZ gains in children with uncomplicated acute malnutrition. Overall, the RUTF+rice bran treated children had greater growth metrics during the treatment period, and the differences between the PP and ITT analysis may be explained by the exclusion of participants who withdrew before the study´s completion in week 4. No adverse events were reported in either treatment arm, and no significant difference in RUTF treatment compliance was observed. Therefore, rice bran is considered an acceptable and tolerable food ingredient for inclusion in RUTFs moving forward.

Weight gain (in both RUTF arms) was greatest in the first four weeks of treatment, and the RUTF+rice bran treatment group had significantly greater weight gain velocity when compared to the RUTF without rice bran. It is possible that the enhanced nutrient density derived from rice bran contributed to this rate increase, as previously demonstrated in a study providing rice bran supplementation in Malian infants.^(31)^ While the weight gain velocity was less than the anticipated 2.0–5.0 g/kg/day, these findings are similar to those of other studies in Asia.^(35,38,50)^ Rachmadewi et al. tested weight gain and product acceptability of a standard RUTF compared to novel RUTF recipes in Indonesia, including those with locally available ingredients such as soybean, mungbean, or peanuts (including the same RUTF as used in the present study) in the treatment of SAM. The study found that the standard peanut-based RUTF and soybean RUTF had the greatest weight gain at >2.0 g/kg/day, while the locally produced peanut-based RUTF had an average weight gain velocity of 1.6 g/kg/day over the eight-week intervention period, but this difference was not statistically significant.^(35)^ Other studies in Cambodia and Vietnam found weight gain velocities in children with SAM treated with both standard and locally produced RUTFs ranging between 1.06 and 1.71 g/kg/day.^(38,50)^ Hence, weight gain in the outpatient treatment of uncomplicated malnutrition is lower than WHO recommendations in many of these countries around Asia. Notably, the present study showed that rice bran addition to the RUTF led to more rapid weight gain during the treatment period. This is in line with a study in Bangladesh in which children with MAM aged 12–18 months receiving a microbiota-directed complementary food arm showed a greater change in WAZ and WHZ than those in the control group.^(21)^ The first two years of life are a critical window of opportunity to include pre- or probiotics in the treatment of acute malnutrition, as rapid maturation occurs in the gut microbiome, as well as regulation of metabolic, neural, and immune pathways. However, children in the 24–59-month group demonstrated stronger growth effects during the first four weeks of treatment. This could be attributed to food patterns, metabolic development differences, different physical demands, and potentially a more mature gut microbiota compared in the 24–59-month children compared to the 6–23-month children. Such characteristics may have allowed the older group to more effectively utilise the rice bran for growth during malnutrition recovery.

The results of the present study indicate that the RUTF+rice bran effectively improves MUAC and weight during the first four weeks of treatment but that these positive effects were not sustained over the follow-up period. The study found no significant difference in MUAC between the two treatment groups in the ITT analysis during or after the treatment period. To calculate MUAC velocity, a PP analysis was applied due to the need to compare each child to their baseline data. In the PP analysis, when measuring MUAC velocity according to PP analysis (Table 2), the RUTF+rice bran group demonstrated a statistically significant greater increase in MUAC in the first four weeks of treatment. Over the eight weeks of treatment, MUAC increased by 0.77 (0.08 SE) mm per week in the RUTF+rice bran group and by 0.61 (0.06 SE) mm per week in RUTF (Table 2). RUTF+rice bran demonstrated a higher increase in MUAC compared to a study conducted in Cambodia that found an average increase of 0.63 mm/week and 0.35 mm/week for a standard RUTF and a locally produced RUTF respectively.^(50)^ Our increases in MUAC are also higher as reported by study in Vietnam, which found an average increase in MUAC of 0.5 (± 0.3 SD) cm over the 4-week intervention period,^(38)^ but these children were not diagnosed with acute malnutrition. Given that both the RUTF and RUTF+rice bran treatment groups increased in MUAC across all age groups during the treatment period, which was not maintained post-treatment, we put forth that continued nutritional interventions during follow-up care are necessary for acutely malnourished children to sustain the gain in MUAC.

Adding rice bran enhanced amino acid and fibre intake for these children, possibly contributing to better growth and reduced inflammation. Complementary feeding studies in Malawi,^(51)^ Mali,^(31,52)^ and Nicaragua^(25)^ support these findings, showing that legumes and rice bran improve growth in infants. In Nicaragua, consumption of rice bran improved several health outcomes, including the gut mucosal lining, decreased gut inflammation, and reduced intestinal permeability while improving serum GLP-2 levels, acting as an antimicrobial,^(26)^ by inhibiting the growth of pathogenic bacteria and reducing intestinal permeability while improving serum GLP-2 levels.^(31,53)^ These effects of rice bran on the gut microbiome and intestinal lining may have also promoted anthropometric growth in the RUTF+rice bran group during the treatment period. This is crucial for growth, especially in the first few years of life.^(54)^

Our study had several strengths alongside the rigour of a double-blinded, randomised, controlled trial design. Importantly, investigators remained blinded during data analysis to prevent post-trial bias. Furthermore, the home visits and data collection were performed by Indonesian field workers with an associate degree in nutrition and experience in research and fieldwork in diverse settings. All staff were fluent in at least one local language used by the families in the study. The fieldworkers performed weekly home visits and could be reached through WhatsApp at any time, which helped keep participants and their families engaged and committed for the entire 16 weeks of the study.

Limitations of this study included underestimated dropout rates in the power calculations, with high dropout rates at week 4 of treatment due to children not gaining enough weight to maintain enrolment per national health protocols, resulting in 22% of children dropping out of the study. However, after week 4, no other participants defaulted from the study. Furthermore, the power calculations were performed for the entire cohort analysis and did not take into account sub analyses of children split by age groups. Compliance in consuming the intended dose of RUTF in the SEHAT clinical trial was 20.94% and 21.23% on average for RUTF and RUTF+rice bran, which can be considered as low; however, it was similar to other studies around Asia.^(50,55)^ Importantly, this clinical trial exemplified a real-life setting, and consumption was not as strictly enforced as in a clinic site. Though the RUTF flavour was changed at week 4 (from chocolate to vanilla, or vice versa) to prevent boredom, compliance rates remained low in both treatment arms. Low compliance was likely related to poorer growth rates and weight gain, as evidenced in this clinical trial. Another limitation is the single country setting of the study, and it remains to be determined how the results may translate to other regions.

## Conclusions

This clinical trial provides the first evidence for the use of stabilised rice bran incorporated into products in acute malnutrition treatment. Here it was found that the RUTF+rice bran significantly improved weight gain and MUAC velocity when compared to RUTF without rice bran. These findings have significant implications for the treatment of malnourished children. We must also specifically highlight the importance of considering age (above and below 2 years old) when designing treatment programmes and the need for follow-up interventions to support recovery and prevent relapse in growth of malnourished children. Even after children have completed the standard treatment programmes, they remain vulnerable to malnutrition impacts long term. Malnutrition affects a child’s physical and cognitive development, and follow-up interventions should ensure sustained growth for optimal multi-system development. Incorporating rice bran in RUTFs demonstrated beneficial effects on ponderal and linear growth. Furthermore, as Indonesia is the third largest rice producer in the world, rice bran is readily available for supplementation in community public health programmes. Rice bran could provide an affordable, nutrient-dense food ingredient in the treatment of malnutrition and regular dietary consumption in the follow up period. These study outcomes warrant contribution to ongoing government efforts that seek to scale up acute malnutrition outpatient programmes with a locally produced RUTF and will inform the current policy for product standardisation for the treatment of acute malnutrition.

## Supporting information

Barbazza et al. supplementary material 1 Barbazza et al. supplementary material

Barbazza et al. supplementary material 2 Barbazza et al. supplementary material

Barbazza et al. supplementary material 3 Barbazza et al. supplementary material

Barbazza et al. supplementary material 4 Barbazza et al. supplementary material

Barbazza et al. supplementary material 5 Barbazza et al. supplementary material

Barbazza et al. supplementary material 6 Barbazza et al. supplementary material

Barbazza et al. supplementary material 7 Barbazza et al. supplementary material

Barbazza et al. supplementary material 8 Barbazza et al. supplementary material

## Data Availability

Data described in the manuscript, code book, and analytic code will be made available upon request, pending application and approval by corresponding authors.

## Supplementary material

The supplementary material for this article can be found at https://doi.org/10.1017/jns.2025.10074
