# Supplementary material for: Inclusion of stabilised rice bran in ready-to-use therapeutic food supports growth in Indonesian children with severe and moderate acute malnutrition: solutions to enhance health with alternative treatments (SEHAT), a double-blinded, randomised clinical trial
Source: J Nutr Sci. 2026 Jan 29;15:e13. doi: 10.1017/jns.2025.10074 (PMC12926669; doi:10.1017/jns.2025.10074)
Supplement: Barbazza et al. supplementary material 1 — Barbazza et al. supplementary material [file S2048679025100748sup001.docx]

**Supplemental Table 1.** RUTF Ingredient and Nutritional Profile

|  | **RUTF-Rice Bran** | **RUTF** |
| --- | --- | --- |
| **Ingredients, g/100 g** | | |
| Palm oil | 20.8 | 21.7 |
| Whole milk powder | 18.1 | 17.6 |
| Peanut butter | 8.6 | 8.6 |
| Sugar | 14.4 | 15.0 |
| Whey protein concentrate | 8.0 | 7.9 |
| Skim milk powder | 4.0 | 6.7 |
| Wheat flour | 8.7 | 9.2 |
| Rice flour | 8.4 | 8.7 |
| Maltodextrin | 0.1 | 0.7 |
| Vitamin and minerals premix | 2.0 | 2.0 |
| Rice bran | 5.0 | - |
| Cocoa powder/vanilla | 1.4 | 1.4 |
| Proprietary ingredients (binder) | 0.5 | 0.5 |
| **Nutrient composition, g/100 g** | | |
| Energy, kcal/100g | 520 | 534 |
| Protein | 15.0 | 13.5 |
| Fat | 31.1 | 31.6 |
| Carbohydrate | 44.4 | 49.0 |
| Fibre | 2.0 | 1.5 |
| Protein-Energy Ratio | 11.5 | 10.1 |
| Fat-Energy Ratio | 53.8 | 53.2 |
