# Supplementary material for: Inclusion of stabilised rice bran in ready-to-use therapeutic food supports growth in Indonesian children with severe and moderate acute malnutrition: solutions to enhance health with alternative treatments (SEHAT), a double-blinded, randomised clinical trial
Source: J Nutr Sci. 2026 Jan 29;15:e13. doi: 10.1017/jns.2025.10074 (PMC12926669; doi:10.1017/jns.2025.10074)
Supplement: Barbazza et al. supplementary material 2 — Barbazza et al. supplementary material [file S2048679025100748sup002.docx]

| **Supplemental Table 2.** Nutritional analysis of SN100, Stabil Nutrition | | | | | | |
| --- | --- | --- | --- | --- | --- | --- |
| **Nutrient** | **Unit** | | **per 100 g** | | | **Method** |
| **PROXIMATES** | | | | | | |
| Protein | g | | 16.12 | | | AOAC 991.20.1 |
| Total Fat | g | | 19.52 | | | AOAC 960.39 |
| Total Carbohydrate | g | | 49.20 | | | Calculation |
| Dietary Fiber | g | | 21.57 | | | AOAC 991.43 |
| Soluble Fiber | g | | 4.48 | | | AOAC 991.43 |
| Insoluble Fiber | g | | 17.09 | | | AOAC 991.43 |
| Ash | g | | 8.45 | | | AOAC 925.51A |
| Moisture | g | | 6.68 | | | ASTA 2.1 |
| Calories | kcal | | 360 | | | Calculation |
| **MICRONUTRIENTS** | | | | | | |
| Thiamin, B1 | | mg | | 2.35 | AOAC 942.23 | |
| Riboflavin, B2 | | mg | | 0.44 | AOAC 970.65 | |
| Niacin, B3 | | mg | | 46.8 | AOAC 944.13 | |
| Pantothenic acid, B5 | | mg | | 1.93 | AOAC 960.46 & Kit | |
| Pyridoxin, B6 | | mg | | 0.54 | AOAC 960.46 & Kit | |
| Folate, B9 | | mcg | | 98.2 | AOAC 960.46 & Kit | |
| Tocopherols | | mg | | 12 | AACC 86-06 | |
| Tocotrienols | | mg | | 13 | AACC 86-06 | |
| Beta carotene | | IU | | ND | CIFSTJ 1982 15:16 | |
| Sodium | | mg | | 8 | AOAC 984.27 | |
| Potassium | | mg | | 1570 | AOAC 984.27 | |
| Calcium | | mg | | 40 | AOAC 984.27 | |
| Magnesium | | mg | | 730 | AOAC 984.27 | |
| Phosphorus | | mg | | 1590 | AOAC 984.27 | |
| Iron | | mg | | 8 | AOAC 984.27 | |
| Phytic Acid | | % | | 5.62 | Analytical Biochemistry Vol. 77:536-539 (1977) | |
