# Supplementary material for: Inclusion of stabilised rice bran in ready-to-use therapeutic food supports growth in Indonesian children with severe and moderate acute malnutrition: solutions to enhance health with alternative treatments (SEHAT), a double-blinded, randomised clinical trial
Source: J Nutr Sci. 2026 Jan 29;15:e13. doi: 10.1017/jns.2025.10074 (PMC12926669; doi:10.1017/jns.2025.10074)
Supplement: Barbazza et al. supplementary material 3 — Barbazza et al. supplementary material [file S2048679025100748sup003.docx]

**Supplemental Table 3**. RUTF consumption dose according to children’s body weight for SEHAT RCT

| **Child’s Weight (Kg)** | **Sachet per day** | **Sachet per week** | **Kcal per day** |
| --- | --- | --- | --- |
| 4.0-4.9 | 1 ½ | 10 | 750 |
| 5.0-6.9 | 2 | 15 | 1,000 |
| 7.0-9.9 | 3 | 20 | 1,500 |
| 10.0-14.9 | 4 | 30 | 2,000 |
