# Supplementary material for: Inclusion of stabilised rice bran in ready-to-use therapeutic food supports growth in Indonesian children with severe and moderate acute malnutrition: solutions to enhance health with alternative treatments (SEHAT), a double-blinded, randomised clinical trial
Source: J Nutr Sci. 2026 Jan 29;15:e13. doi: 10.1017/jns.2025.10074 (PMC12926669; doi:10.1017/jns.2025.10074)
Supplement: Barbazza et al. supplementary material 4 — Barbazza et al. supplementary material [file S2048679025100748sup004.docx]

| **Supplemental Table 4**. Average percent RUTF consumption **week 1-8, 1-4, and 5-8** by treatment and age group. | | | | | | | | | | | | | | | |
| --- | --- | --- | --- | --- | --- | --- | --- | --- | --- | --- | --- | --- | --- | --- | --- |
|  | **All ages** | | | | | **6-23m** | | | | | **24-59m** | | | | |
| **Treatment** | **N** | **Mean** | **Sd** | **SE** | **p-value** | **N** | **Mean** | **Sd** | **SE** | **p-value** | **N** | **Mean** | **Sd** | **SE** | **p-value** |
| **Week 1-8** | | | | | | | | | | | | | | | |
| RUTF+rice bran | 74 | 21.23 | 17.26 | 2.01 | 0.91 | 26 | 24.63 | 16.38 | 3.21 | 0.23 | 48 | 19.38 | 17.62 | 2.54 | 0.45 |
| RUTF | 82 | 20.94 | 14.22 | 1.57 |  | 35 | 19.72 | 14.55 | 2.46 |  | 47 | 21.85 | 14.06 | 2.05 |  |
| **Week 1-4** | | | | | | | | | | | | | | | |
| RUTF+rice bran | 75 | 23.60 | 18.97 | 2.19 | 0.85 | 25 | 29.11 | 20.37 | 4.07 | 0.19 | 50 | 20.84 | 17.80 | 2.52 | 0.22 |
| RUTF | 82 | 24.15 | 17.16 | 1.89 |  | 35 | 22.59 | 16.52 | 2.79 |  | 47 | 25.31 | 17.71 | 2.58 |  |
| **Week 5-8** | | | | | | | | | | | | | | | |
| RUTF+rice bran | 74 | 18.31 | 17.16 | 1.99 | 0.81 | 26 | 20.04 | 14.90 | 2.92 | 0.40 | 48 | 17.38 | 18.35 | 2.65 | 0.76 |
| RUTF | 82 | 17.73 | 13.08 | 1.44 |  | 35 | 16.86 | 13.78 | 2.33 |  | 47 | 18.38 | 12.64 | 1.84 |  |
