# Supplementary material for: Inclusion of stabilised rice bran in ready-to-use therapeutic food supports growth in Indonesian children with severe and moderate acute malnutrition: solutions to enhance health with alternative treatments (SEHAT), a double-blinded, randomised clinical trial
Source: J Nutr Sci. 2026 Jan 29;15:e13. doi: 10.1017/jns.2025.10074 (PMC12926669; doi:10.1017/jns.2025.10074)
Supplement: Barbazza et al. supplementary material 5 — Barbazza et al. supplementary material [file S2048679025100748sup005.docx]

| **Supplemental Table 5.** Participants graduated and or recovered from SAM or MAM at each time point, by treatment group. | | | |
| --- | --- | --- | --- |
| **Recovery** | **RUTF^1^**  **n=105** | **RUTF+ rice bran^1^**  **n=95** | **p-value^2^** |
| Graduated^3^ | 34  (41%) | 36  (49%) | 0.33 |
| Graduated^4^ from SAM at week 8 | 21  (22%) | 24  (27%) | 0.42 |
| Recovered ^5^ from MAM at week 8 | 2  (2.2%) | 5  (6.2%) | 0.25 |
| Graduated^4^ from SAM at week 12 | 22  (23%) | 19  (22%) | 0.80 |
| Recovered^5^  from MAM at week 12 | 3  (3.3%) | 1  (1.2%) | 0.62 |
| Graduated^4^ from SAM at week 16 | 19  (20%) | 19  (22%) | 0.79 |
| Recovered^5^  from MAM at week 16 | 5  (5.4%) | 4  (4.9%) | >0.99 |
| Recovered^6^ from SAM at week 8 | 2  (2.1%) | 1  (1.1%) | >0.99 |
| Recovered^6^ from SAM at week 12 | 2  (2.1%) | 4  (4.5%) | 0.43 |
| Recovered^6^ from SAM at week 16 | 2  (2.1%) | 3  (3.4%) | 0.67 |
| ^1^ n (%)  ^2^ Pearson’s Chi-squared test; Fisher’s exact test  ^3^ SAM or MAM at enrollment graduated to MAM or fully recovered (defined as: WHLZ >= -2 and or MUAC>= 12.5) at week 8, 12 or 16  ^4^ SAM at enrollment graduated to MAM  ^5^ Enrolled as MAM and recovered (defined as: WHLZ >= -2 and or MUAC>= 12.5).  ^6^ Enrolled as SAM and recovered (defined as: WHLZ >= -2 and or MUAC>= 12.5). | | | |
