# Supplementary material for: Inclusion of stabilised rice bran in ready-to-use therapeutic food supports growth in Indonesian children with severe and moderate acute malnutrition: solutions to enhance health with alternative treatments (SEHAT), a double-blinded, randomised clinical trial
Source: J Nutr Sci. 2026 Jan 29;15:e13. doi: 10.1017/jns.2025.10074 (PMC12926669; doi:10.1017/jns.2025.10074)
Supplement: Barbazza et al. supplementary material 6 — Barbazza et al. supplementary material [file S2048679025100748sup006.docx]

**Supplemental Table 6.** Primary outcomes, weight, weight-for-height z-score, height-for-age z-score, weight-for-age z-score, and mid-upper arm circumference assessment from the mixed linear models.

|  |  | **All ages** | | | | | **6-23 months of age** | | | | | **24-59 months of age** | | | | |
| --- | --- | --- | --- | --- | --- | --- | --- | --- | --- | --- | --- | --- | --- | --- | --- | --- |
| **Assessment** | **Visit** | **RUTF** | ***n*** | **RUTF+rice bran** | ***n*** | ***p-*value**  **Test of Interaction** | **RUTF** | ***n*** | **RUTF**  **+rice bran** | ***n*** | ***p-*value**  **Test of Interaction** | **RUTF** | ***n*** | **RUTF**  **+rice bran** | ***n*** | ***p-*value**  **Test of Interaction** |
| Weight, kg | Baseline | 8.49 (0.19) | 105 | 8.72 (0.18) | 95 | **0.020** | 6.82 (0.12) | 43 | 7.06 (0.15) | 35 | 0.484 | 9.65  (0.21) | 62 | 9.70 (0.17) | 60 | **0.032** |
|  | Week 4 | 8.93 (0.23) | 82 | 9.18  (0.20) | 75 |  | 7.12 (0.14) | 35 | 7.47 (0.19) | 25 |  | 10.27 (0.24) | 47 | 10.03 (0.19) | 50 |  |
|  | Week 8 | 9.02 (0.23) | 82 | 9.20  (0.20) | 74 |  | 7.20  (0.15) | 35 | 7.60 (0.20) | 26 |  | 10.38 (0.24) | 47 | 10.07 (0.20) | 48 |  |
|  | Week 12 | 9.14  (0.23) | 82 | 9.22  (0.19) | 75 |  | 7.34 (0.15) | 35 | 7.68  (0.21) | 26 |  | 10.48 (0.25) | 47 | 10.03  (0.19) | 49 |  |
|  | Week 16 | 9.25  (0.22) | 82 | 9.34  (0.20) | 74 |  | 7.51  (0.15) | 35 | 7.81  (0.21) | 26 |  | 10.54 (0.24) | 47 | 10.16 (0.20) | 48 |  |
| Weight-for-height *z-*score | Baseline | -3.03 (0.03) | 105 | -3.06 (0.04) | 95 | 0.780 | -3.07  (0.05) | 43 | -3.07  (0.07) | 35 | 0.630 | -3.00 (0.05) | 62 | -3.06 (0.06) | 60 | **0.014** |
|  | Week 4 | -2.75 (0.05) | 82 | -2.68  (0.07) | 75 |  | -2.79  (0.08) | 35 | -2.76  (0.13) | 25 |  | -2.72 (0.06) | 47 | -2.64 (0.08) | 50 |  |
|  | Week 8 | -2.75  (0.05) | 82 | -2.71  (0.07) | 74 |  | -2.84 (0.08) | 35 | -2.71  (0.14) | 26 |  | -2.69 (0.07) | 47 | -2.71  (0.07) | 48 |  |
|  | Week 12 | -2.78  (0.05) | 82 | -2.86 (0.07) | 75 |  | -2.85 (0.08) | 35 | -2.76 (0.14) | 26 |  | -2.73 (0.07) | 47 | -2.91 (0.07) | 49 |  |
|  | Week 16 | -2.77  (0.05) | 82 | -2.83 (0.07) | 74 |  | -2.79  (0.08) | 35 | -2.73  (0.13) | 26 |  | -2.76 (0.07) | 47 | -2.88 (0.08) | 48 |  |
| Weight-for-age *z-*score | Baseline | -3.48 (0.05) | 105 | -3.59 (0.06) | 95 | 0.103 | -3.49 (0.08) | 43 | -3.57 (0.10) | 35 | 0.533 | -3.48 (0.07) | 62 | -3.61 (0.08) | 60 | 0.087 |
|  | Week 4 | -3.23 (0.07) | 82 | -3.36 (0.08) | 75 |  | -3.25 (0.11) | 35 | -3.32 (0.15) | 25 |  | -3.22 (0.08) | 47 | -3.38 (0.09) | 50 |  |
|  | Week 8 | -3.26 (0.07) | 82 | -3.38 (0.08) | 74 |  | -3.32 (0.11) | 35 | -3.34 (0.16) | 26 |  | -3.22 (0.08) | 47 | -3.40 (0.09) | 48 |  |
|  | Week 12 | -3.26 (0.07) | 82 | -3.46 (0.08) | 75 |  | -3.30 (0.10) | 35 | -3.39 (0.15) | 26 |  | -3.23 (0.09) | 47 | -3.50 (0.08) | 49 |  |
|  | Week 16 | -3.25 (0.06) | 82 | -3.44 (0.08) | 74 |  | -3.25 (0.11) | 35 | -3.37 (0.15) | 26 |  | -3.24 (0.08) | 47 | -3.47 (0.09) | 48 |  |
| Height-for-age *z-*score | Baseline | -2.60 (0.09) | 105 | -2.78 (0.10) | 95 | 0.389 | -2.58 (0.15) | 43 | -2.76 (0.18) | 35 | 0.759 | -2.60 (0.12) | 62 | -2.80 (0.12) | 60 | 0.329 |
|  | Week 4 | -2.46 (0.10) | 82 | -2.75 (0.11) | 75 |  | -2.52 (0.16) | 35 | -2.67 (0.22) | 25 |  | -2.42 (0.14) | 47 | -2.80 (0.13) | 50 |  |
|  | Week 8 | -2.51 (0.10) | 82 | -2.76 (0.11) | 74 |  | -2.60 (0.16) | 35 | -2.73 (0.20) | 26 |  | -2.45 (0.14) | 47 | -2.78 (0.14) | 48 |  |
|  | Week 12 | -2.47 (0.10) | 82 | -2.74 (0.11) | 75 |  | -2.55 (0.15) | 35 | -2.76 (0.21) | 26 |  | -2.40 (0.14) | 47 | -2.74 (0.14) | 49 |  |
|  | Week 16 | -2.46 (0.10) | 82 | -2.75 (0.11) | 74 |  | -2.54 (0.16) | 35 | -2.76 (0.20) | 26 |  | -2.40 (0.14) | 47 | -2.74 (0.14) | 48 |  |
| Mid-upper arm circumference, cm | Baseline | 12.77 (0.07) | 105 | 12.78 (0.08) | 95 | 0.153 | 12.33 (0.11) | 43 | 12.30 (0.12) | 35 | 0.805 | 13.07 (0.07) | 62 | 13.05 (0.08) | 60 | 0.118 |
|  | Week 4 | 13.19 (0.09) | 82 | 13.35 (0.10) | 75 |  | 12.72 (0.12) | 35 | 12.75 (0.16) | 25 |  | 13.53 (0.10) | 47 | 13.65 (0.10) | 50 |  |
|  | Week 8 | 13.26 (0.09) | 82 | 13.39 (0.10) | 74 |  | 12.78 (0.12) | 35 | 12.84 (0.17) | 26 |  | 13.61 (0.10) | 47 | 13.69 (0.11) | 48 |  |
|  | Week 12 | 13.24 (0.08) | 82 | 13.29 (0.09) | 75 |  | 12.77 (0.12) | 35 | 12.82 (0.17) | 26 |  | 13.59 (0.09) | 47 | 13.53 (0.10) | 49 |  |
|  | Week 16 | 13.24 (0.08) | 82 | 13.28 (0.10) | 74 |  | 12.80 (0.13) | 35 | 12.83 (0.18) | 26 |  | 13.58 (0.09) | 47 | 13.52 (0.11) | 48 |  |

Data are reported as means (standard error). Statistical significance level p < 0.05. The linear mixed model fixed effects include treatment, week (0, 4, 8, 12, 16), treatment*week interactions, age, sex and child ID as a random effect.
