# Supplementary material for: Inclusion of stabilised rice bran in ready-to-use therapeutic food supports growth in Indonesian children with severe and moderate acute malnutrition: solutions to enhance health with alternative treatments (SEHAT), a double-blinded, randomised clinical trial
Source: J Nutr Sci. 2026 Jan 29;15:e13. doi: 10.1017/jns.2025.10074 (PMC12926669; doi:10.1017/jns.2025.10074)
Supplement: Barbazza et al. supplementary material 7 — Barbazza et al. supplementary material [file S2048679025100748sup007.docx]

| **Supplemental Table 7.** Intention-to-treat analyses of primary outcomes weight and weight-for-height z-score. Pairwise comparison of treatment arms by week from mixed linear models were adjusted with Bonferroni correction. | | | | | | | | | | | | | | | | | | | | | | |
| --- | --- | --- | --- | --- | --- | --- | --- | --- | --- | --- | --- | --- | --- | --- | --- | --- | --- | --- | --- | --- | --- | --- |
|  |  | **All ages** | | | | | | | | **6-23 months of age** | | | | | | | | **24-59 months of age** | | | | |
| **Outcome** | **Contrast** | **Estimate** | **SE** | **df** | | **t.ratio** | | **p-value** | | **Estimate** | | **SE** | | **df** | **t.ratio** | **p-value** | | **Estimate** | **SE** | **df** | **t.ratio** | **p-value** |
| Weight | RUTF+rice bran – RUTF week 0 | -0.149 | 0.100 | 218.893 | | -1.496 | | 0.680 | | -0.049 | 0.124 | | 85.292 | | -0.399 | | 1 | -0.221 | 0.141 | 130.560 | -1.573 | 0.590 |
|  | RUTF+rice bran – RUTF week 4 | -0.075 | 0.102 | 236.943 | | -0.742 | | 1 | | -0.018 | 0.128 | | 96.127 | | -0.143 | | 1 | -0.130 | 0.143 | 139.700 | -0.909 | 1 |
|  | RUTF+rice bran – RUTF week 8 | -0.077 | 0.102 | 237.375 | | -0.756 | | 1 | | 0.058 | 0.128 | | 95.481 | | 0.452 | | 1 | -0.175 | 0.144 | 140.379 | -1.217 | 1 |
|  | RUTF+rice bran – RUTF week 12 | -0.193 | 0.102 | 236.974 | | -1.900 | | 0.295 | | -0.004 | 0.128 | | 95.481 | | -0.034 | | 1 | -0.315 | 0.143 | 140.057 | -2.195 | 0.150 |
|  | RUTF+rice bran – RUTF week 16 | -0.181 | 0.102 | 237.387 | | -1.774 | | 0.385 | | -0.044 | 0.128 | | 95.481 | | -0.345 | | 1 | -0.262 | 0.144 | 140.390 | -1.823 | 0.350 |
| Weight-for-height *z-*score | RUTF+rice bran – RUTF week 0 | -0.045 | 0.070 | | 308.273 | | -0.644 | | 1 | -0.043 | 0.117 | | 117.342 | | -0.366 | | 1 | -0.070 | 0.088 | 183.126 | -0.793 | 1 |
|  | RUTF+rice bran – RUTF week 4 | 0.072 | 0.075 | | 370.751 | | 0.956 | | 1 | 0.022 | 0.127 | | 146.687 | | 0.175 | | 1 | 0.075 | 0.094 | 219.276 | 0.798 | 1 |
|  | RUTF+rice bran – RUTF week 8 | 0.045 | 0.075 | | 372.459 | | 0.602 | | 1 | 0.114 | 0.126 | | 144.598 | | 0.906 | | 1 | -0.021 | 0.095 | 222.476 | -0.224 | 1 |
|  | RUTF+rice bran – RUTF week 12 | -0.076 | 0.075 | | 370.717 | | -1.021 | | 1 | 0.076 | 0.126 | | 144.598 | | 0.605 | | 1 | -0.189 | 0.094 | 220.703 | -2.006 | 0.230 |
|  | RUTF+rice bran – RUTF week 16 | -0.044 | 0.075 | | 372.488 | | -0.583 | | 1 | 0.044 | 0.126 | | 144.598 | | 0.349 | | 1 | -0.110 | 0.095 | 222.943 | -1.160 | 1 |
| Statistical significance level *p <* 0.05. | | | | | | | | | | | | | | | | | | | | | | |
