# Supplementary material for: Inclusion of stabilised rice bran in ready-to-use therapeutic food supports growth in Indonesian children with severe and moderate acute malnutrition: solutions to enhance health with alternative treatments (SEHAT), a double-blinded, randomised clinical trial
Source: J Nutr Sci. 2026 Jan 29;15:e13. doi: 10.1017/jns.2025.10074 (PMC12926669; doi:10.1017/jns.2025.10074)
Supplement: Barbazza et al. supplementary material 8 — Barbazza et al. supplementary material [file S2048679025100748sup008.docx]

| **Supplemental Table 8.** Per-protocol analyses of weight-for-height z-score, weight-for-age z-scores, and height-for-age z-scores compared to baseline. Pairwise comparison of treatment arms by week from mixed linear models were adjusted with Bonferroni correction. | | | | | | | | | | | | | | | | | | | | | |
| --- | --- | --- | --- | --- | --- | --- | --- | --- | --- | --- | --- | --- | --- | --- | --- | --- | --- | --- | --- | --- | --- |
|  |  | **All ages** | | | | | | | | **6-23 months of age** | | | | | | | **24-59 months of age** | | | | |
| **Outcome** | **Contrast** | **Estimate** | **SE** | **df** | | **t.ratio** | | **p-value** | | **Estimate** | | **SE** | | **df** | **t.ratio** | **p-value** | **Estimate** | **SE** | **df** | **t.ratio** | **p-value** |
| Weight-for-height *z-*score | RUTF+rice bran – RUTF week 4 | -0.120 | 0.067 | 296.834 | | -1.798 | | 0.292 | | -0.002 | 0.118 | | 104.201 | | -0.019 | 1 | -0.134 | 0.081 | 184.516 | -1.658 | 1 |
|  | RUTF+rice bran – RUTF week 8 | -0.095 | 0.067 | 298.218 | | -1.421 | | 0.624 | | -0.094 | 0.117 | | 102.690 | | -0.804 | 1 | -0.039 | 0.081 | 187.423 | -0.481 | 1 |
|  | RUTF+rice bran – RUTF week 12 | 0.026 | 0.067 | 296.645 | | 0.396 | | 1 | | -0.056 | 0.117 | | 102.690 | | -0.480 | 1 | 0.125 | 0.081 | 185.929 | 1.542 | 0.500 |
|  | RUTF+rice bran – RUTF week 16 | -0.007 | 0.067 | 298.236 | | -0.102 | | 1 | | -0.024 | 0.117 | | 102.690 | | -0.204 | 1 | 0.044 | 0.081 | 187.409 | 0.548 | 1 |
| Weight-for-age z-score | RUTF+rice bran – RUTF week 4 | -0.066 | 0.042 | | 279.622 | | -1.565 | | 0.476 | -0.004 | 0.081 | | 99.223 | | -0.049 | 1 | -0.084 | 0.048 | 167.986 | -1.746 | 0.332 |
|  | RUTF+rice bran – RUTF week 8 | -0.084 | 0.042 | | 280.917 | | -2.000 | | 0.118 | -0.086 | 0.081 | | 97.848 | | -1.064 | 1 | -0.061 | 0.049 | 170.561 | -1.252 | 0.848 |
|  | RUTF+rice bran – RUTF week 12 | -0.003 | 0.042 | | 279.495 | | -0.071 | | 1 | -0.022 | 0.081 | | 97.848 | | -0.277 | 1 | 0.027 | 0.048 | 169.277 | 0.558 | 1 |
|  | RUTF+rice bran – RUTF week 16 | -0.011 | 0.042 | | 280.933 | | -0.258 | | 1 | 0.014 | 0.081 | | 97.848 | | 0.167 | 1 | -0.012 | 0.049 | 170.547 | -0.255 | 1 |
| Height-for-age z-score | RUTF+rice bran – RUTF week 4 | -0.007 | 0.039 | | 272.461 | | -0.185 | | 1 | 0.020 | 0.091 | | 91.915 | | 0.223 | 1 | -0.017 | 0.033 | 189.686 | -0.495 | 1 |
|  | RUTF+rice bran – RUTF week 8 | -0.063 | 0.039 | | 273.714 | | -1.615 | | 0.788 | -0.056 | 0.091 | | 90.752 | | -0.611 | 1 | -0.054 | 0.034 | 192.679 | -1.609 | 0.436 |
|  | RUTF+rice bran – RUTF week 12 | -0.039 | 0.039 | | 272.359 | | -1.006 | | 1 | 0.021 | 0.091 | | 90.752 | | 0.235 | 1 | -0.059 | 0.034 | 191.127 | -1.772 | 0.312 |
|  | RUTF+rice bran – RUTF week 16 | -0.024 | 0.039 | | 273.729 | | -0.610 | | 1 | 0.036 | 0.091 | | 90.752 | | 0.394 | 1 | -0.045 | 0.034 | 192.665 | -1.336 | 0.732 |
|  |  |  |  |  |  |  |  |  |  |  |  |  |  |  |  |  |  |  |  |  |  |
|  | | | | | | | | | | | | | | | | | | | | | |
